# Use of Ultra-Hydrophilic Absorbable Polysaccharide for Bleeding Control in Cardiothoracic Surgical Procedures

**DOI:** 10.3390/medicina61020230

**Published:** 2025-01-27

**Authors:** Dow Rosenzweig, Peter Lamm, Christoph Schmitz, Ferdinand Vogt

**Affiliations:** 1 Department of Cardiac Surgery, Artemed Klinikum München Süd, 81379 Munich, Germany; dow.rosenzweig@artemed.de (D.R.); peter.lamm@artemed.de (P.L.); 2 Department of Cardiac Surgery, University of Munich, 81377 Munich, Germany; chr.schmitz@me.com; 3 Cardiovascular Research Unit, Chris Barnard Division of Cardiothoracic Surgery, University of Cape Town, Cape Town 7700, South Africa; 4 Department of Cardiac Surgery, Paracelsus Medical University Nuremberg, 90419 Nurnberg, Germany

**Keywords:** Starsil^®^ Hemostat, bleeding control, plant-based microporous polysaccharide, polysaccharide, hemostatic powder, cardiac surgery

## Abstract

*Background and Objectives:* Operative blood loss is strongly correlated with morbidity and mortality in surgery. Various hemostatic agents are used to reduce bleeding in cardiothoracic procedures. We report our experience with a plant-based microporous polysaccharide hemostatic powder (Starsil^®^ Hemostat, Hemostat Medical GmbH, Velen, Germany). *Materials and Methods:* Data were collected retrospectively from 65 patients who underwent cardiac surgery at our institution from January 2012 to January 2015 with (*n* = 42) or without (*n* = 23; control group) the use of the hemostat powder. Primary endpoints were safety (e.g., laboratory parameters, adverse events, and infection parameters) and time to hemostasis when the hemostat powder was used. Other endpoints included operation time, hospitalization, quantity of the hemostat powder applied, and length of stay in the intensive care unit. *Results:* The 65 patients (49 male:16 female) analyzed in the study underwent 65 cardiothoracic procedures, including off-pump coronary artery bypass grafts (*n* = 25), on-pump coronary artery bypass grafts (*n* = 6), valve procedures (*n* = 6), valve procedures in combination with bypass grafts (*n* = 7), and others (*n* = 21). The application of the hemostat powder did not increase adverse events. The laboratory parameters did not exceed the expected range after heart surgery in both groups. The hemostat powder had no significant impact on the laboratory parameters compared to the control group. Blood control was sufficient and was rated by surgeons from good to very good on a visual analog scale (VAS) from 1 (very bad) to 10 (very good) [VAS = 8.3 ± 1.2]. Intraoperative hemostasis was possible in nearly all patients. The hemostat powder led to satisfactory bleeding control within 2 min in 88% of cases. Five patients needed a second 5 g unit of the hemostat powder. *Conclusions:* The observed parameters between groups did not differ significantly. Therefore, the use of Starsil^®^ Hemostat in cardiothoracic surgery is safe and effective bleeding control was achieved.

## 1. Introduction

Perioperative bleeding control is a crucial issue during cardiac procedures. To prevent or control bleeding, sutures and electrocautery are primarily used alongside precise surgical techniques. Nevertheless, significant intraoperative bleeding can lead to prolonged operating room times, increased reliance on blood transfusions, and, in severe cases, a heightened risk of adverse events or mortality [1]. In case conventional hemostatic techniques with sutures or electrocautery are ineffective, various hemostatic agents can be used as an adjunct to control or reduce bleeding in cardiothoracic procedures [2,3]. However, it is crucial to acknowledge that some of these hemostatic products may carry the risk of adverse effects [4,5]. Topical hemostatic agents are typically applied directly to the bleeding site. This study focuses on the clinical application and outcomes associated with the hemostat powder, which is a plant-derived microporous polysaccharide powder used as a topical agent to control perioperative bleeding during cardiothoracic surgeries.

## 2. Materials and Methods

### 2.1. Patient Population

The data were routinely collected during routine internal quality control checks to assess the efficacy and safety of medical products in patients who underwent cardiothoracic surgery at our institution between January 2012 and January 2015. Medical products for bleeding control were used for complex cardiothoracic surgery or surgery with prolonged hemostasis at the surgeon’s discretion. Of 65 patients in the database, the hemostat powder was used to control bleeding in 42 patients. These patients were compared to 23 patients who received other methods of bleeding control (e.g., Ostene^®^ (Baxter, Unterschleißheim, Germany) for hemostasis on the sternum after sternotomy, oxidized regenerated cellulose, Tachosil^®^ (Takeda Austria GmbH, Linz, Austria), or fibrin glue). The other products were also used where Starsil^®^ Hemostat (Hemostat Medical GmbH, Velen, Germany) was not recommended according to the instructions for use (IFU). Only patients with routine indications were included in the data analysis. Data from emergency patients or patients with preoperatively proven bacterial colonization in the preoperatively performed control or patients with preoperative infections were excluded. Demographic patent data are listed in Table 1. There were no significant changes in technique or protocol in the performance of the operations during the study period. The main focus of this study was the safety of use, the performance of the hemostat powder on local hemostasis, and the surgeons’ satisfaction with the product. Surgeons rated the performance of the hemostat powder, e.g., for bleeding control and in terms of the handling of the device, using a visual analog scale (VAS) from 1 to 10, with 1 being very bad and 10 being a perfect product performance. Bleeding control was rated satisfactory if the bleeding stopped within 2 min as described in the IFU. Other study goals were laboratory values such as hemoglobin, leucocytes, C-reactive protein, creatinine, and blood glucose. In addition, the temperature of patients was documented. Preoperative values in Table 2 were obtained one day before surgery or on admission. The maximum/minimum values were taken during the inpatient stay (day 3–day 8) and the “discharge” values were determined within the last 24 h before discharge.

### 2.2. Surgical Technique

**Figure 1:**
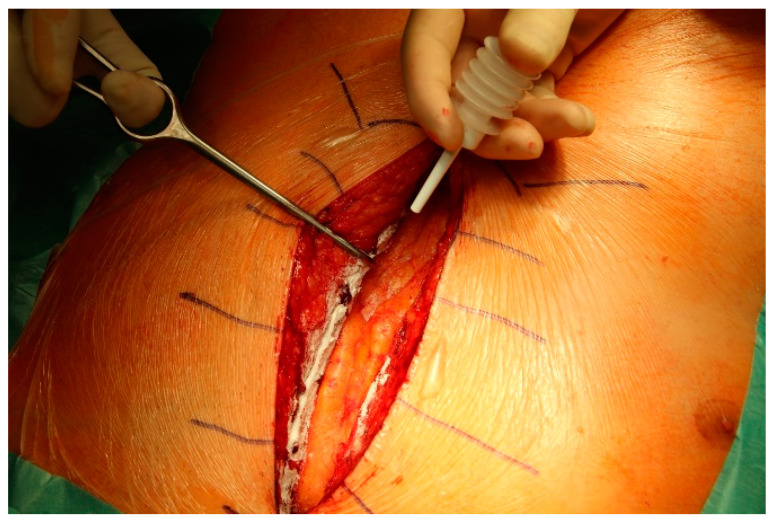
Use of Starsil^®^ Hemostat for bleeding control from the sternum.

**Figure 2:**
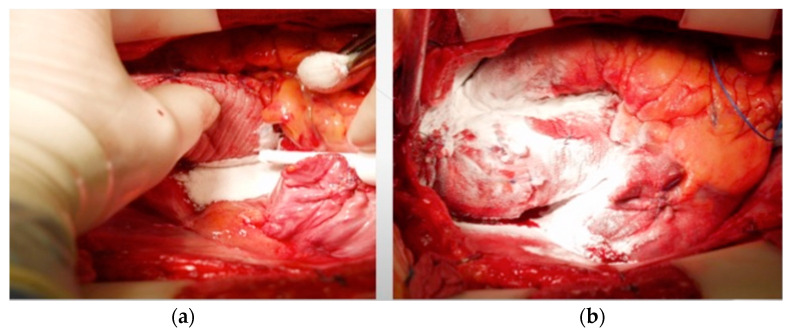
Use of Starsil^®^ Hemostat for bleeding control after replacement of the ascending aorta with a Dacron prosthesis: (**a**) bleeding control behind the aortic prosthesis; (**b**) liberal use of the hemostat powder all over the prosthesis.

**Figure 3:**
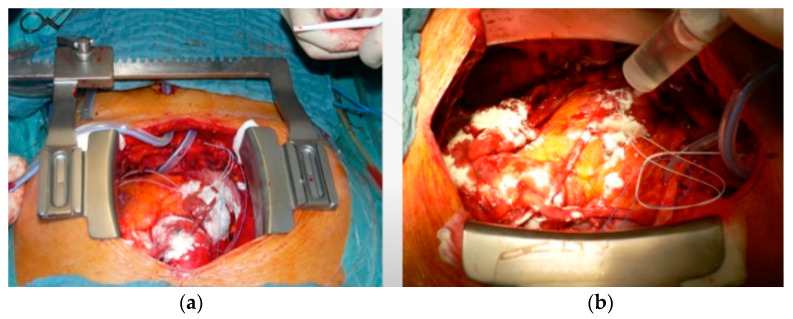
Use of Starsil^®^ Hemostat for bleeding control in surgical myocardial revascularization: (**a**) OPCAB = off-pump coronary artery bypass; (**b**) CABG = coronary artery bypass graft with heart–lung machine.

All operations were performed through full median sternotomy. Heparinization was instituted in all operations. For cardiac operations performed with cardiopulmonary bypass (CPB), an active clotting time (ACT) of >600 s. was aimed for. For off-pump coronary artery bypass (OPCAB) procedures and others without CPB, the ACT had to be >300 s. After weaning from extracorporeal circulation or completion of the anastomoses of the OPCAB operations, heparin was antagonized with protamine sulfate. Further hemostasis of active bleeding sources with sutures and/or electrocautery followed. Patients treated with the hemostat powder to control bleeding received up to 10 g of the hemostatic powder. The hemostat powder was applied to the bleeding sources according to the IFU provided by the manufacturer (Figure 1, Figure 2 and Figure 3). The number of applications or the amount of the hemostat powder used depended on the level of bleeding and was the surgeon’s decision. Before sternal closure, residual powder was applied to both sides of the sternum (Figure 1). The sternum was closed with sternal wires, subcutaneous fat, and skin with absorbable sutures.

### 2.3. Study Product

Starsil^®^ Hemostat (Hemostat Medical GmbH, Velen, Germany) is a class III medical device that is intended to be used in surgery as an adjunctive hemostat to control capillary, arterial, or venous bleeding in situations where the use of ligatures, pressure, or other conventional methods prove to be inadequate or impracticable. The device is indicated for adhesion prevention as well in cavities covered by mesothelium. Starsil^®^ Hemostat is a hemostat consisting of 5 g of a purified plant-based absorbable polysaccharide that can be administered to the entire operation area. The powder is available off-the-shelf without any further preparation. In order to obtain hemostasis, it can be applied directly to a bleeding wound. The hemostatic effect results from rapid dehydration and subsequent concentration of blood components like red blood cells, platelets, and serum proteins (thrombin, fibrinogen, etc.), thus accelerating the clotting cascade. As a result, a gelled adhesive matrix is produced. Normal platelet activation and fibrin deposition produce a clot that functions as a mechanical barrier and limits further bleeding. The absorption of the particles is achieved within approximately 48 to 72 h. The hemostat powder is biocompatible, non-pyrogenic, and contains no allo- or xenogenic additions.

### 2.4. Statistical Methods

Data were retrospectively entered into a computerized database and analyzed through SPSS software version 11.0.1 for Windows under the guidance of a statistician. Continuous data are presented as the mean +/− standard deviation. The results are given as mean values with the standard deviation of the mean values within the population. The α-level is 5%, whereby values with *p* < 0.05 are considered significant.

## 3. Results

### Outcome

Laboratory parameters were collected from all patients (Table 2). The average age of the entire patient group was 70.2 years. At the time of surgery, the patients with the hemostat powder were between 47 and 84 years old, and those without were 58–84 years old. The average hospital stay was 12.9 days. The patients in whom the hemostat powder was used during surgery did not have significantly shorter hospitalization times (12.6 ± 2.0 vs. 13.1 ± 2.7 days; *p* = 0.933). There were no intraoperative or postoperative deaths in either group. There was one reoperation due to postoperative bleeding in each group. In the Starsil group, the identified cause was bleeding due to inadequate coagulation of a subcostal artery of the mammary artery bed. In the control group, a side branch of the mammary artery graft had to be clipped to stop the bleeding. In both groups, there were no deep or major sternal wound infections that required re-intervention or led to prolonged hospitalization. All but two patients were routinely discharged from the hospital to a rehabilitation center. Two patients refused rehabilitation. In the Starsil group, 97.6% (*n* = 41 patients) were followed up in our medical center during the first six months; in the group without the hemostat powder, the figure was 95.6% (*n* = 22 patients). One patient in each group did not want the follow-up examination at our center but at their family doctor. During the follow-up of the patients up to 6 months postoperatively, none of the patients had died. No adverse events attributable to Starsil^®^ Hemostat or to any other medical product were reported during the postoperative follow-up period.

As seen in Table 2, Starsil^®^ Hemostat had no significant impact on the laboratory parameters. Hemoglobin and creatinine did not significantly differ between groups. Preoperative blood glucose (mg/dl) was significantly higher in the group that underwent surgery without the hemostat powder (108.9 ± 31.0 vs. 144.0 ± 24.2; *p* = 0.008). The postoperative maximum value was also significantly higher in the group without the hemostat powder (134.0 ± 18.5 vs. 177.5 ± 22.6; *p* = 0.003). Before discharge, the value was not significantly higher (103.9 ± 25.23 vs. 130.4 ± 25.2; *p* = 0.055).

The infection parameters obtained perioperatively are listed in Table 2. But in detail, the CRP preop (Table 2) was not significantly slightly higher in the Starsil group than in the patients in whom Starsil was not used (0.83 ± 0.45 vs. 0.62 ± 0.28; *p* = 0.458). The same applies to CRP max. It was not significantly higher in the Starsil group (19.63 ± 8.09 vs. 19.13 ± 4.13, *p* = 0.924). Even before discharge, the value was not significantly higher in the Starsil group (4.9 ± 2.38 vs. 3.63 ± 1.08, *p* = 0.286). For the leukocytes (Table 2), the preoperative average values were within the normal range and were not significantly different between the groups (7.25 ± 0.58 vs. 7.86 ± 0.84; *p* = 0.206). For the postoperative maximum values, the leukocyte values were not significantly lower in the Starsil group (12.85 ± 1.38 vs. 15.12 ± 2.96; *p* = 0.109) and were back in the normal range in both groups before discharge (7.91 ± 0.72 vs. 7.70 ± 0.84; *p* = 0.704). The postoperative maximum body temperature (Table 2) was not significantly higher in the Starsil group than in the control group (37.3 ± 0.33 vs. 36.8 ± 0.38; *p* = 0.075). Before discharge, the body temperature was back in normal range in both groups (36.8 ± 0.09 vs. 36.6 ± 0.22; *p* = 0.175).

In the Starsil group, satisfactory bleeding control was achieved in all but one case. One surgeon rated a second application and the use as “non-satisfactory”. The hemostat led to satisfactory bleeding control within 2 min in 88% of the patients with a 5 g unit. Five patients needed a second application of the hemostat using another 5 g unit for bleeding control. On average, bleeding control was achieved in 94 ± 56 s. In general, the surgeon’s satisfaction according to the VAS was 8.3 ± 1.2 (Table 1).

## 4. Discussion

The primary aim of this study was to evaluate the safety and efficacy of Starsil^®^ Hemostat based on the data collected for our clinic’s quality assurance program. Hemostasis was achieved in the predicted time of less than 2 min according to the IFU in most cases and with high satisfaction of the surgeons. This fact is supported by other studies using polysaccharide hemostatic powders. Li [6] reported achieving hemostasis in a mean of 110 s. No adverse events occurred in their study or our study. The laboratory parameters in our study remained within the expected range. Shifts to abnormal values were typical for the surgical interventions performed and showed no significant differences compared to the control group, except for the blood glucose. Values were already higher in the control group. Limitations of the study are mainly the retrospective character of the study, a highly selected and heterogeneous patient group, short follow-up duration, and, of course, the small study population. In addition, due to the blinded data set, potentially essential data such as the number of required blood units could not be collected retrospectively. However, bleeding control is an integral part of all cardiac surgeries, and one of the study’s primary objectives was to ensure the safety of this medical product. In the context of the available literature [7,8,9,10,11], in which the hemostat powder was used in cardiac surgery and other procedures, the present study supports using the polysaccharide powder as a safe adjunct to surgical bleeding control.

Bruckner has demonstrated that the use of starch powder results in significant improvements when performing complex cardiothoracic procedures [12]. In his retrospective study, he observed 240 patients from January 2009 to January 2013 with (*n* = 103) or without (*n* = 137) the use of a polysaccharide absorbable hemostat powder. It led to a significant reduction in hemostasis time compared to an untreated control group (hemostat 93.4 ± 41 min vs. control 107.6 ± 56 min; *p* = 0.02), postoperative chest tube output, and need for postoperative blood transfusion. Furthermore, a non-significantly shorter ICU length of stay (*p* = 0.08) was observed in the observation group. Although 30-day mortality was not significantly different, these results confirm both the clinical benefits of starch powders and also indicate potential economic benefits by reducing blood transfusions and length of stay. Boucher [13] reported that the economic impact of bleeding is significant, as it is directly related to increased resource consumption in hospitals. The extent of bleeding correlates directly with increased costs, e.g., surgical re-interventions. For instance, the costs in cardiac surgery can rise dramatically, as much as EUR 30,000.

Surgeons have a high acceptance of hemostatic powders as an adjunct in hemostasis. A European multidisciplinary expert group in hemostasis and hemostatics confirmed the positive benefits of hemostatic powders in surgical practice in a consensus paper [2]. Questionnaires were answered by 79 high-volume surgeons from various surgical fields regarding the use of hemostatic powders, and 95% of statements regarding hemostatic powders were rated as important or very important. However, this paper also confirmed that the hemostat should not be a substitute for good surgical technique and the appropriate use of electrocautery, sutures, clips, and staples. The proper use and handling of hemostatic products may effectively support bleeding control during surgery, as recently confirmed in the Cochrane Database of Systematic Reviews [14]. The meta-analysis included 24 trials with 2376 participants undergoing vascular surgery. The sealant used demonstrated a reduction in time to hemostasis. In our study and the literature, almost no adverse events were reported with starch powders. However, it is crucial to use the right hemostatic agent for the adequate surgical procedure in an appropriately timed fashion, not only to improve clinical outcomes and avoid adverse events, but also to limit the overall cost of treatment.

Furthermore, in a study [15] with the hemostatic polysaccharide Perclot, adverse events were reported when using the powder during cardiac rhythm device implantation, such as CRP increase and pain. However, there are some shortcomings of the study: the use of Perclot was only described rudimentarily in the manuscript—the important step of deactivating excess Perclot with saline after hemostasis was not carried out, as well as specific product training. However, the instructions for use make specific reference to this. Powder that has not been deactivated is often still very active and can, e.g., lead to pH shifts and the dehydration of the wound, leading to aggressive reactions. In contrast, none of these adverse events were observed in another study by House [16]. In the prospective randomized multicenter study (19 centers) with 324 patients undergoing open elective cardiac, general, or urologic surgery, hemostasis was performed with two starch powders (Arista^®^ and Perclot^®^) equivalent to Starsil^®^. In this study, 161 patients were treated with Perclot^®^ and 163 with Arista^®^ and monitored for up to six weeks postoperatively. No disadvantage of Perclot^®^ compared to Arista^®^ was found. No safety concerns were identified. This impressively demonstrates how crucial the correct indication for the product group and the appropriate application according to IFU is.

In our study, other hemostatic products were used in the control group, and no product-related adverse events were reported either. The literature highlights their effectiveness in reducing blood loss and hematoma formation. However, significant risks and side effects are also noted. For instance, in a review, Masoudi [17] discusses the complications associated with oxidized regenerated cellulose, which continue to emerge; above all, these include granulomas, abscesses, hematomas, cysts, hemorrhagic complications, and masses misdiagnosed as tumors, as well as pain and infections. Other frequently used hemostats include products made from porcine gelatin, bovine collagen, human-pooled plasma thrombin, or mixtures of bovine gelatin and human-pooled plasma thrombin, among others. While their hemostatic efficacy is well-established, they are associated with considerable costs and serious complications, such as anaphylactic reactions, infection nidus formation, compression of local structures (e.g., nerves or vessels), delayed wound healing, product dislodgement, granulomas, inflammation, or neurotoxicity [18,19,20]. In cardiac surgery, one of the most commonly used hemostats is Tachosil^®^ (Takeda Pharmaceutical Company Limited, Tokyo, Japan), which is composed of human fibrinogen and thrombin combined with equine collagen. It is a porous patch that is activated with saline and adheres effectively. Few side effects have been reported, including hypersensitivity to human proteins and the risk of ileus in abdominal surgeries. Unfortunately, it is expensive and limited to local applications. Moreover, its application in hard-to-reach areas is challenging, and it is nearly impossible to use in thoracoscopic procedures, making it unsuitable for minimally invasive surgeries. In contrast, Starsil^®^ Hemostat powder can be applied over larger areas of the surgical field, particularly in difficult-to-access regions or through endoscopic instruments.

## 5. Conclusions

Starsil^®^ Hemostat is a safe and effective adjunctive for hemostasis during cardiac surgery. There were no unusual perioperative changes in laboratory parameters or body temperature. No adverse in-hospital or post-discharge events were attributed to the use of the powder. This hemostatic powder may fasten hemostasis, decrease transfusion, and reduce operation time. Further and more extensive studies are needed to confirm this.

## Figures and Tables

**Table 1:** Patient demographics and perioperative data.

	Total Population	Patients with Starsil^®^ Hemostat	Patients Without Starsil^®^ Hemostat	*p* Value *
number (*n*)	65	42	23	
age (years)	70.2	68.9 ± 3.04	72.8 ± 4.73	0.125
gender (m/f)	49/16	31/11	18/5	n.s.
weight (kg)	85.7	85.5 ± 9.8	85.9 ± 14.7	0.957
height (cm)	174.2	176.8 ± 5.9	170.9 ± 5.9	0.137
Type of operation (*n*)				
OPCAB	25	14	12	
CABG	6	4	2	
AVR	4	3	1	
MVR/MV-repair	2	2	0	
AVR + CABG	6	5	1	
MVR + CABG	1	1	0	
Aortic surgery	3	2	1	
Other *	18	11	7	
Starsil^®^ Hemostat (g)	-	5.4 ± 2.8	0	
time to hemostasis [with Starsil^®^ Hemostat (s)]	-	94 ± 56	-	
Surg. satisfaction (VAS)	-	8.3 ± 1.2	-	
Duration of OP (min)	158.5	153.1 ± 54.5	164.7 ± 66.5	0.770
Ventilation (*n*)	42	34	18	-
Ventilation time (h)	21.5	21.6 ± 9.2	21.2 ± 10.7	0.954
ICU (h)	90.1	88.2 ± 40.0	93.8 ± 32.5	0.830
Hospitalization (Tage)	12.9	12.6 ± 2.0	13.1 ± 2.7	0.933

*n*: number, X ± x: mean values with standard deviation, m/f: male/female, OPCAB: off-pump coronary bypass, CABG: coronary artery bypass graft, AVR: aortic valve replacement, MVR/MV-Repair: mitral valve replacement/mitral valve repair, Other *: e.g., additional pacemaker implantation or additional ablation, “time to hemostasis”: time to hemostasis from application of Starsil Hemostat to termination of bleeding, s: seconds, VAS: visual analog scale with 1 as very bad and 10 as very good, OP: operation, min: minutes, h: hours, ICU: intensive care unit, *p*-value *: significance level with *p* < 0.05 is significant and *p* ≥ 0.05 or n.s. = not significant.

**Table 2:** Results of laboratory parameters.

	OP with Starsil^®^ Hemostat			OP Without Starsil^®^ Hemostat			*p* Value
Hb preop	14.1	±	0.75	13.5	±	0.70	0.325
Hb mini	9.3	±	0.75	8.8	±	0.62	0.383
Hb pre-discharge	11.3	±	0.72	10.6	±	0.57	0.184
Crea preop	1.13	±	0.17	1.12	±	0.18	0.904
Crea max	1.29	±	0.22	1.30	±	0.26	0.924
Crea pre-discharge	1.13	±	0.16	1.11	±	0.21	0.883
Glu preop	108.9	±	31.0	144.0	±	24.2	0.008
Glu max	134.0	±	18.5	177.5	±	22.6	0.003
Glu pre-discharge	103.9	±	25.23	130.4	±	25.2	0.055
Infection parameters							
CRP preop	0.83	±	0.45	0.62	±	0.28	0.458
CRP max	19.63	±	8.09	19.13	±	4.13	0.924
CRP pre-discharge	4.9	±	2.38	3.63	±	1.08	0.286
Leucocytes preop	7.25	±	0.58	7.86	±	0.84	0.206
Leucocytes max	12.85	±	1.38	15.12	±	2.96	0.109
Leuc. pre-discharge	7.91	±	0.72	7.70	±	0.84	0.704
Temp preop	36.7	±	0.17	36.6	±	0.19	0.400
Temp max	37.3	±	0.33	36.8	±	0.38	0.075
Temp pre-discharge	36.8	±	0.09	36.6	±	0.22	0.175

X ± x: mean values with standard deviation, OP: operation, preop: preoperatively, max: maximum value recorded after the operation during hospitalization, mini: minimum value recorded after the operation during hospitalization, pre-discharge: value of the last laboratory sample taken before discharge, Hb: hemoglobin (g/dL), Crea: creatine (mg/dL), Glu: serum glucose (g/dL), CRP: C-reactive protein (mg/L), Leuc.: Leucocytes Temp: temperature (°C), *p*-value = significance level with *p* < 0.05 is significant and *p* ≥ 0.05 = not significant.

## Data Availability

The raw data supporting the conclusions of this article will be made available by the authors on request.
